# Supplementary material for: High Rates of Multidrug-Resistant Escherichia coli in Great Cormorants (Phalacrocorax carbo) of the German Baltic and North Sea Coasts: Indication of Environmental Contamination and a Potential Public Health Risk
Source: Pathogens. 2022 Jul 27;11(8):836. doi: 10.3390/pathogens11080836 (PMC9416030; doi:10.3390/pathogens11080836)
Supplement: Supplementary file 1 [file pathogens-11-00836-s001.zip › pathogens-1804239-supplementary.pdf]

Sample	Location	Growth on AB plates	no. of AB plates with bacterial growth	Ampicillin	Cephalotin	Chloramphenicol	Ciprofloxacin	Colistin	Gentamycin	Sulfasoxazole	Tetracyclin	*E. coli* isolated
**1**	Wallnau	yes	1	no	no	no	no	yes	no	no	no	yes
**2**	Wallnau	yes	3	no	no	yes	no	yes	no	yes	no	yes
**3**	Wallnau	yes	6	yes	yes	yes	no	no	yes	yes	yes	yes
**4**	Wallnau	yes	2	no	yes	no	no	yes	no	no	no	yes
**5**	Wallnau	yes	1	no	no	no	no	yes	no	no	no	yes
**6**	Wallnau	yes	1	no	no	no	no	yes	no	no	no	no
**7**	Wallnau	yes	2	no	no	no	no	yes	no	no	yes	yes
**8**	Wallnau	yes	3	no	no	no	no	yes	no	yes	yes	yes
**9**	Wallnau	yes	3	no	yes	no	no	no	no	yes	yes	yes
**10**	Wallnau	no	0	no	no	no	no	no	no	no	no	x
**11**	Wallnau	yes	1	no	no	no	no	yes	no	no	no	yes
**12**	Sylt	yes	6	yes	yes	yes	no	yes	no	yes	yes	yes
**13**	Friedrichsgabekoog	yes	5	yes	yes	yes	no	yes	no	no	yes	no
**14**	Friedrichsgabekoog	yes	5	yes	yes	no	no	yes	no	yes	yes	yes
**15**	Friedrichsgabekoog	yes	6	yes	yes	yes	no	yes	no	yes	yes	yes
**16**	Friedrichsgabekoog	yes	2	no	no	no	no	yes	no	no	yes	no
**17**	Friedrichsgabekoog	yes	1	no	no	no	yes	no	no	no	no	yes
**18**	Friedrichsgabekoog	no	0	no	no	no	no	no	no	no	no	x
**19**	Friedrichsgabekoog	no	0	no	no	no	no	no	no	no	no	x
**20**	Friedrichsgabekoog	yes	1	no	no	no	no	yes	no	no	no	yes
**21**	Friedrichsgabekoog	yes	2	no	yes	no	no	yes	no	no	no	yes
**22**	Friedrichsgabekoog	yes	7	yes	yes	no	yes	yes	yes	yes	yes	yes
**23**	Friedrichsgabekoog	yes	2	no	no	yes	no	yes	no	no	no	no
**24**	Friedrichsgabekoog	yes	6	yes	no	yes	yes	yes	no	yes	yes	yes

**25**	Geltinger Birk	yes	7	yes	yes	yes	yes	yes	yes	no	yes	yes
**26**	Geltinger Birk	yes	4	yes	yes	no	no	yes	no	no	yes	yes
**27**	Geltinger Birk	yes	6	yes	yes	no	yes	yes	yes	no	yes	yes
**28**	Geltinger Birk	yes	5	yes	yes	yes	no	yes	no	no	yes	no
**29**	Geltinger Birk	no	0	no	no	no	no	no	no	no	no	x
**30**	Geltinger Birk	yes	5	yes	yes	yes	no	yes	no	no	yes	no
**31**	Geltinger Birk	yes	7	yes	yes	yes	yes	yes	no	yes	yes	yes
**32**	Geltinger Birk	yes	5	yes	yes	yes	yes	yes	no	no	no	no
**33**	Geltinger Birk	yes	4	yes	yes	yes	no	yes	no	no	no	yes

Supplementary Table S1. Antimicrobial plates. The table shows all cormorant sample numbers and which sample grew on which antibiotic containing plate as well if *E. coli* was isolated from at least one of the plates. No *E. coli* isolation was performed in table cells with x because of missing bacterial growth on all antibiotic containing agar plates. All samples showed bacterial growth either on Blood agar, Gassner agar or in the Mossel bouillon indicating bacterial presence in the samples.

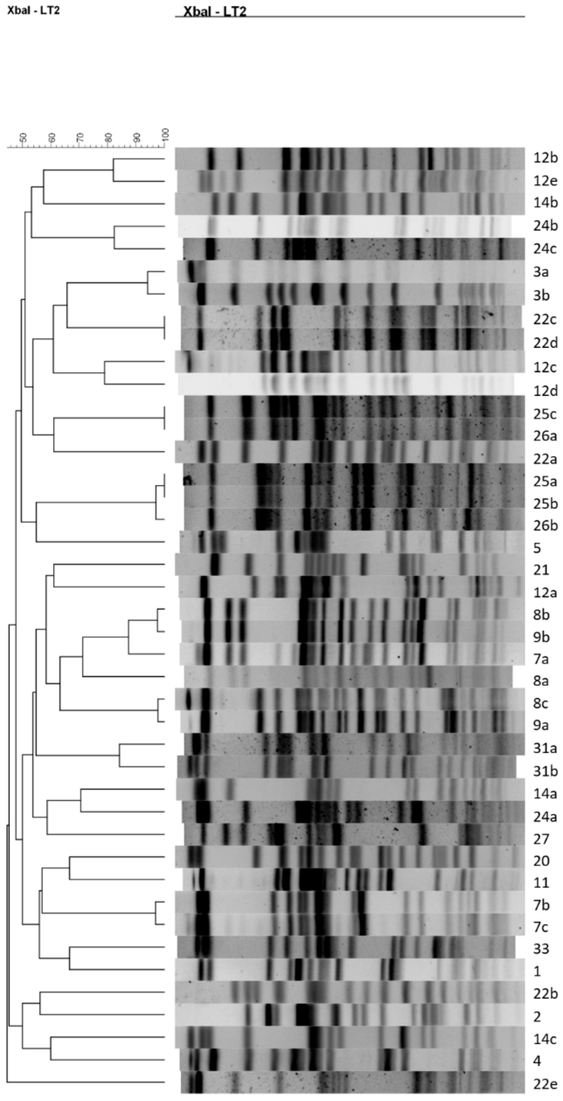

Supplementary Figure S1. The figure shows the dendrogram including the pulsed-field gel electrophoresis band patterns of the 42 isolates that were typeable by XbaI macrorestriction.
